# Synthesis of Linear Geranylphenols and Their Effect on Mycelial Growth of Plant Pathogen *Botrytis cinerea*

**DOI:** 10.3390/molecules19021512

**Published:** 2014-01-27

**Authors:** Luis Espinoza, Lautaro Taborga, Katy Díaz, Andrés F. Olea, Hugo Peña-Cortés

**Affiliations:** 1 Departamento de Química, Universidad Técnica Federico Santa María, Av. España No. 1680, Valparaíso 2340000, Chile; E-Mails: lautaro.taborga@usm.cl (L.T.); katy.diaz@usm.cl (K.D.); 2 Departamento de Ciencias Químicas, Facultad de Ciencias Exactas, Universidad Andrés Bello, Quillota 980, Viña del Mar 2520000, Chile; E-Mail: aolea@unab.cl; 3 Dirección de Investigación, Universidad de Valparaíso, Blanco 951, Valparaíso 2340000, Chile; E-Mail: hugo.pena@uv.cl

**Keywords:** synthesis, linear geranylphenols, antifungal activity, *Botrytis cinerea*

## Abstract

Natural geranyl compounds are known to exhibit important biological activities. In this work a series of geranylphenols were synthesized to evaluate their effect on the mycelial growth of *Botrytis cinerea*. Geranyl derivatives were synthesized by direct geranylation reactions between the corresponding phenol derivatives and geraniol, using BF_3_^.^OEt_2_ as catalyst and AgNO_3_ as secondary catalyst. Previously reported molecules [geranylhydroquinone (**2**), geranylhydroquinone diacetate (**6**) and geranylphloroglucinol (**9**)], and new substances [(*E*)-4-(3,7-dimethylocta-2,6-dienyl)benzene-1,2,3-triol (geranyl-pyrogallol, **7**), (*E*)-4-(3,7-dimethylocta-2,6-dienyl)benzene-1,2,3-triyl triacetate (**8**), (*E*)-2-(3,7-dimethylocta-2,6-dienyl)benzene-1,3,5-triyl triacetate geranylphloroglucinol triacetate (**10**), 2,4-bis((*E*)-3,7-dimethylocta-2,6-dienyl)benzene-1,3,5-triyl triacetate (**11**), 2,6-bis((*E*)-3,7-dimethylocta-2,6-dienyl)-3,5-dihydroxyphenyl acetate (**12**)], were obtained. All compounds were characterized by IR, HRMS and NMR spectroscopic data. The inhibitory effect of the synthesized compounds on the mycelial growth of *Botrytis cinerea* was tested *in vitro.* Excepting compound **11**, all substances constrained the mycelial growth of *Botrytis cinerea.* The antifungal activity depends on the chemical structure of geranylphenol derivatives. Compounds **2** and **9** were the more effective substances showing inhibition degrees higher than those obtained with the commercial fungicide Captan, even at lower concentrations. Monosubstitution on the aromatic nucleus by a geranyl chain seems to be more effective for the inhibition of mycelial growth than a double substitution. These results suggest that the new derivatives of geranylphenols have the ability to block the mycelial development of the plant pathogen *B. cinerea* and that this capacity depends strongly on the structural features and lipophilicity of the compounds.

## 1. Introduction

**Figure 1:**
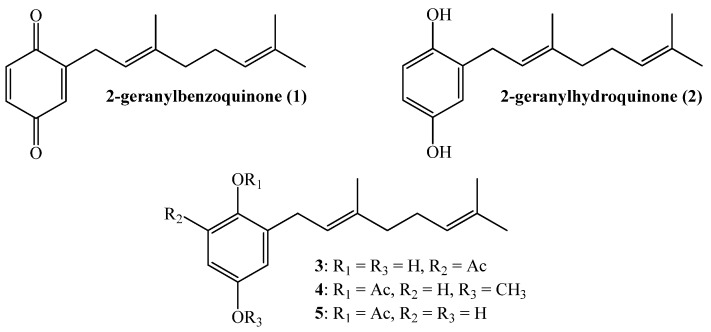
Structure of some active linear geranylphenols.

Several factors highlight the urgent need for the development of new control strategies for the phytopathogenic fungus *Botrytis cinerea* and other plagues affecting important agricultural crops. They include the emergence of highly resistant strains of *B. cinerea* to fungicides, contamination of soil and water, high cost of chemical control and the limited availability of fungicides [1]. In addition, the strong worldwide trend towards sustainable development and environmentally friendly substances should be considered [2]. It has been extensively documented that multiple metabolites, obtained from species belonging to the plant kingdom, have special biological properties suitable for controlling several types of animal and plant pathogens. For instance, linear geranylquinones or geranylhydroquinones, present in higher plants and in marine urochordates [3], exhibit cytotoxic activity and inhibit larval growth and development. Some particular compounds such as 2-geranylbenzoquinone (**1**), isolated from *Ascindian Synoicum castellatum* [4], 2-geranylhydroquinone (**2**) isolated from the *Cordia alliodora* tree [2], *Phacelia crenulata* [5,6,7], *Aplidium antillense* [8] and the tunicate *Amaroucium multiplicatum* [9], have been related to biological activities including toxicity, cytotoxicity, antimicrobial, anti-cancer protective and antioxidant effects, among others [6,9,10,11,12,13]. Additionally, linear geranylmethoxyphenol/acetates (compounds **3**–**5**, see Figure 1), isolated from *Phaceliaixodes* [8], are cytotoxic, allergenic and insecticidal, and topical application of 100 µg of geranylbenzoquinone on pupae of *Tenebrio* caused severe abnormalities and death [14].

Several marine sesquiterpenoid quinones and hydroquinones are of considerable scientific interest because of their versatile biological activities where a particular (hydro)quinone may display multiple activities [15].

In previous studies we have reported the synthesis and cytotoxic activity of compounds **1**–**2** and some geranylmethoxyphenol/acetate analogs [16,17]. Considering the activity against phytophagous insects and pathohens of compounds **3** and **4** [14] and assuming the presence of this property in other geranylphenol analogs, we have recently reported the synthesis and structure determination of 14 linear geranylphenols molecules [16].

**Figure 2:**
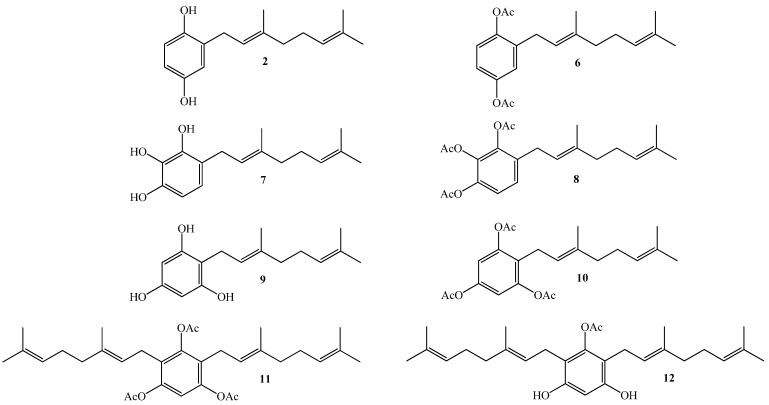
Structure of linear geranylphenols **2**, **6**–**12** synthesized in this work.

Thus, our interest is to get a broader insight into the relationship between the molecular structure and the biological activity against plant pathogens of geranylquinone and geranylhydroquinone derivatives and geranylphenols. We believe that this knowledge might be useful to provide new substances to control such kinds of pathogens. Therefore, in this research we report the synthesis, structure determination, and *in-vitro* antifungal effect against phytopathogenic fungus *Botrytis cinerea* of a series of geranylphenols derivatives **2**, **6**–**12**, (see Figure 2). These compounds were obtained by a modification of a previously reported synthetic method [12,14,16], which consisted in the use of acetonitrile as a solvent instead of dioxane. Also we have evaluated the use of AgNO_3_ as secondary catalyst.

## 2. Results and Discussion

### 2.1. Chemistry

Compound **2** was prepared by using the previously described coupling between geraniol and hydroquinone [16,17,18], but using acetonitrile instead of dioxane as solvent and assessing the use of AgNO_3_ as secondary catalyst. In this case, the compound **2** was obtained with a 32% yield, which is slightly higher than that previously reported by us [16], but similar to that reported by other authors [19]. Compound **6** was obtained in 92.8% yield from compound **2** by a standard acetylation reaction using Ac_2_O and DMAP in CH_2_Cl_2_. The spectroscopic data of compounds **2** and **6** were consistent with those previously reported [5,6,8,20].

**Figure 3:**
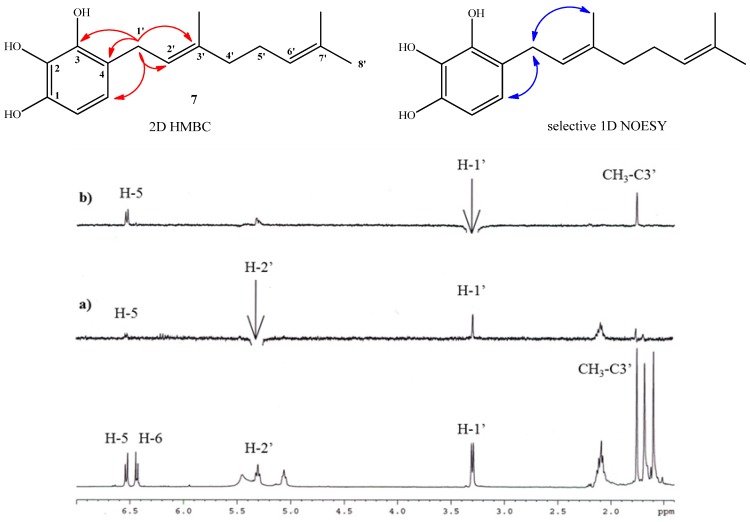
Top: mayor 2D ^1^H-^13^C HMBC and sel. 1D NOESY correlations for compound **7**. Bottom: standard ^1^H-NMR spectrum; (**a**) selective irradiation NOESY at 5.31ppm (H-2'). (**b**) selective irradiation NOESY at 3.30 ppm (H-1').

Compound **7** (for which the name geranylpyrogallol is proposed in analogy with the name of geranylphloroglucinol [18,21]) was prepared in 18.1% yield following the same experimental procedure described for the synthesis of compound **2**. The structure of **7** was mainly established by NMR, where aromatic signals at *δ*_H_ = 6.53 (d, *J* = 8.3 Hz, 1H) and *δ*_H_ = 6.44 (d, *J* = 8.3 Hz, 1H) were observed as two doublets for hydrogens H-5 and H-6, confirming the aromatic monosubstitution position. Additionally, in the HMBC spectrum, the signal at *δ*_H_ = 3.30 ppm assigned to H-1' (d, *J* = 7.1 Hz, 2H, H-1') shows ^3^*J*_H-C_ coupling with C-3 (*δ*_C_ = 142.5), C-3' (*δ*_C_ = 138.4 ppm) and C-5 (*δ*_C_ = 120.2 ppm) and ^2^*J*_H-C_ coupling with C-2' and C-4 (*δ*_C_ = 122.2 ppm and 119.5 respectively). These HMBC correlations are shown in Figure 3. In order to establish the *E* geometry of the C-2'-C-3' double bond of the geranyl chain, selective 1D NOESY NMR experiments were recorded for compound **7**. These correlations are shown in Figure 3, where the most important of those correspond to the correlations observed between the H-1' and the hydrogen H-5 and methyl group in the C-3' position.

Compound **8** was obtained from compound **7** in 93% yield by standard acetylation, using Ac_2_O and DMAP in CH_2_Cl_2_. In the ^1^H-NMR spectrum of the triacetylated derivative **8**, three singlets at *δ*_H_ = 2.29, 2.27 and 2.26 ppm (each 3H, CH_3_CO) were observed. Additionally, in the ^13^C-NMR spectrum the signals appearing at *δ*_C_ = 168.0, 167.7 and 167.1 ppm (C=O), confirmed the presence of the triacetylated derivative.

**Scheme 1:**
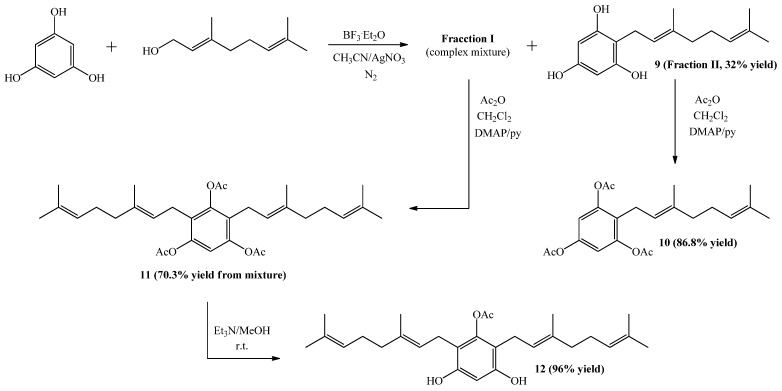
Synthesis of compounds **9**–**12**.

The synthesis of compound **9** from phloroglucinol and geraniol was performed following the experimental procedure described in Scheme 1. However, after completion of reaction two main spots were observed in the TLC analysis. Subsequent column chromatography (C.C.) separation and purification allowed the isolation of the two fractions, a less polar one corresponding to a complex mixture (Fraction I), and a more polar fraction (Fraction II) corresponding to compound **9**, obtained in 32% yield. The spectroscopic data of compound **9** was consistent with that previously reported [18,21].

Subsequently, compound **10** was obtained (in 86.8% yield) from **9** by standard acetylation with Ac_2_O and DMAP in CH_2_Cl_2_. The ^1^H-NMR spectrum of the triacetylated derivative **10** shows two singlet signals at *δ*_H_ = 2.26 ppm (2 × CH_3_CO) and 2.24 ppm (CH_3_CO). Additionally, in the ^13^C-NMR spectrum the signals at *δ*_C_ = 168.6 (2 × C=O) and 168.5 ppm (C=O), confirm the presence of the triacetylated derivative.

**Figure 4:**
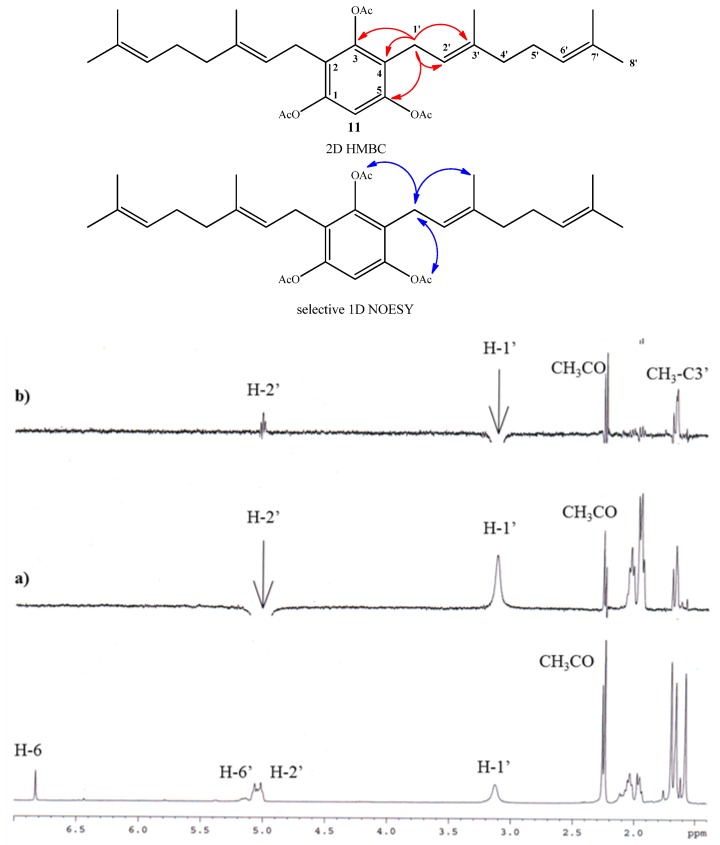
Top: mayor 2D ^1^H-^13^C HMBC and sel. 1D NOESY correlations for compound **11**. Bottom: standard ^1^H-NMR spectrum; (**a**) selective irradiation NOESY at 5.02 ppm (H-2'). (**b**) selective irradiation NOESY at 3.12 ppm (H-1').

Compound **11** was obtained in 70.3% yield by standard acetylation of the complex mixture (Fraction I). Spectroscopic evidence of double substitution on the aromatic nucleus by geranyl chains was established from the ^1^H-NMR spectrum by the following observations: a unique Ar-H signal appears at 6.83 ppm (s, 1H, H-6); and the intensity of this signal was 1:4 relative to the signal at 3.12 ppm (bs, 4H, H-1'). The presence of three acetate groups was evidenced in the ^1^H-NMR spectrum by the observed signals at *δ*_H_ = 2.26 ppm (s, 3H, COCH_3_) and 2.24 ppm (s, 6H, COCH_3_), and corroborated by the presence in the ^13^C-NMR spectrum of signals at *δ*_C_ = 168.6 (2 × COCH_3_) and 168.4 (COCH_3_). Finally, the complete determination of symmetrical structure was established by 2D HMBC and selective 1D NOESY experiments. The major 2D HMBC correlations, which were considered to confirm the structure of compound **11** were a ^3^*J*_H-C_ coupling observed between H-1' with C-3 (*δ*_C_ = 148.6 ppm), with C-1 and C-5 (*δ*_C_ = 147.2 ppm) and C-3' (*δ*_C_ = 135.6 ppm), while ^2^*J*_H-C_ coupling was observed with C-2 and C-4 (*δ*_C_ = 124.6 ppm) and C-2' (*δ*_C_ = 121.0 ppm). From selective 1D NOESY experiments, the *E* geometry in the C-2'-C-3' double bond position of the geranyl chain was established. Here the most important correlations were observed between the H-1' and methyl group in C-3' position and those detected with the acetate groups (Figure 4). The 2D HMBC and 1D NOESY correlations are shown in Figure 4. Additionally, the possible incorporation of two geranyl chains in the aromatic ring was previously described for geranylmethoxyphenol analogues by other authors [22].

**Figure 5:**
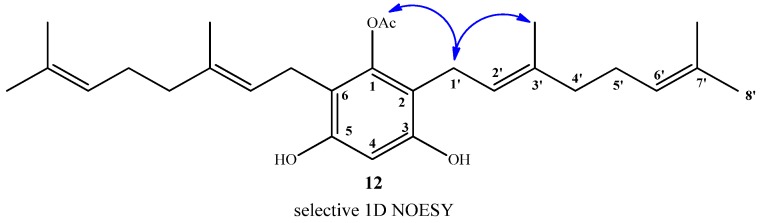
Main selective 1D NOESY correlations for compound **12**.

Finally, compound **12** was obtained in 96% yield by saponification of **11** under mild conditions (Et_3_N/MeOH) at room temperature. The structure of compound **12** was established mainly by comparison of the NMR spectra of compounds **11** and **12**. In the ^1^H-NMR of compound **12**, only one acetate group signal was observed at *δ*_H_ = 2.30 ppm (s, 3H, CH_3_CO), while in the ^13^C-NMR spectrum, only the signal at *δ*_C_ = 169.3 ppm (CO_2_) was observed. The position of the acetyl group in the structure was proposed from the observation of long-range interactions in the 1D NOESY experiments between CH_3_CO and the hydrogens of C-1' (see Figure 5).

On the other hand, the use of AgNO_3_ as secondary catalyst has significant effects on the Friedel-Crafts direct geranylation reaction used to obtain compound **9**, namely, the reaction yield was increased from 4.2% to 32% [20], the formation of compounds disubstituted in the aromatic ring is enhanced, and the degradation of geraniol products is reduced (as evidenced by TLC analysis) and a lower difficulty in the C.C. separation process is observed in all of the cases. This suggests that AgNO_3_ stabilizes geraniol during the reaction process in CH_3_CN solution.

### 2.2. Anti-phytopathogenic Activity against Botrytis cinerea

**Figure 6:**
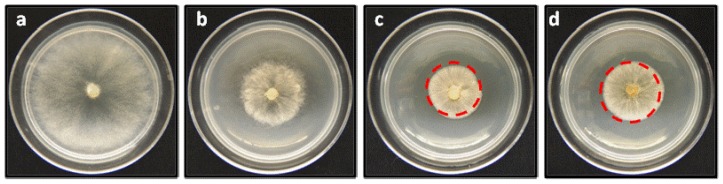
Effect of linear geranylphenols on *in vitro* mycelial growth of *B. cinerea*. (**a**) Negative control, represents a medium containing only PDA; (**b**) positive control, measured in the presence of 250 mg/L of Captan; (**c**) the medium includes compound **2** at 250 mg/L; (**d**) medium contains compound **9** at 250 mg/L.

To evaluate the biological activity of the geranylphenol derivatives **2**, **6**–**12** (Figure 2), their effect on the growth of mycelia of *B. cinerea* was determined *in vitro* after 48 h of incubation by using the agar-diffusion assay technique with PDA as medium. Figure 6 shows a representative example of three independent assays for evaluating the biological activity of the studied compounds. Details of experimental conditions are given in the Experimental section. The results are expressed as percentage of inhibition, which is calculated as the ratio of the area of *B. cinerea* in the presence and absence of geranylphenols, are summarized in Table 1. For comparison, in this table the percentage of inhibition values of captan, a molecule that is widely used to control the growth of *B. cinerea* are included (measured at the same concentration used to determine the activity of geranylphenols).

The data in Table 1 shows that all compounds, with the exception of compound **11**, affect the development of the mycelia of *B. cinerea*, reducing its growth between 56%–86%, compared to the negative control, at concentrations of 150–250 mg/L after 48 h of incubation. Compounds **2** and **9** were the more active ones, showing an inhibitory effect on the mycelial growth that depends on the applied concentration. At 150 mg/L compounds **9** and **2** exhibited inhibitory effects of 66% and 81%, respectively, which increased up to 81% and 86% at higher concentration (250 mg/L). The observed inhibitory capacity exceeds even the value of inhibition obtained with the commercial fungicide captan used as positive control (Table 1). On the other hand, compound **11** seems to be inactive against *B. cinerea*. A grade of the mycelial growth inhibition lower than 50% is observed, independently of the applied concentration.

**Table 1:** Effect of linear geranylphenols and new derivatives on mycelial growth of *B. cinerea.*

Compounds	Percentage of inhibition on *in vitro* mycelial growth of *Botrytis cinerea* (%) *^a^*		
	50 mg/L	150 mg/L	250 mg/L
**2**	58 ± 2.15	82 ± 3.04	86 ± 1.35
**6**	40 ± 7.20	67 ± 1.67	76 ± 1.54
**7**	45 ± 1.36	63 ± 0.65	72 ± 1.78
**8**	28 ± 2.44	56 ± 5.70	64 ± 1.76
**9**	38 ± 0.21	66 ± 7.95	81 ± 2.57
**10**	33 ± 5.48	56 ± 1.80	72 ± 0.87
**11**	0 ± 0.00	16 ± 4.10	26 ± 0.00
**12**	0 ± 0.00	30.4 ± 2.30	66 ± 3.90
C–	0 ± 0.00	0 ± 0.00	0 ± 0.00
Captan	39 ± 7.33	60 ± 8.45	80 ± 2.92

*^a^* The percentage of inhibition of mycelial growth was based on colony diameter measurements after 48 h of incubation. Each point represents the mean of at least three independent experiments ± standard deviation.

Analysis of the results indicates that the antifungal activity depends on the chemical structure of the geranylphenol derivatives. Thus, compounds having hydroxyl groups attached to the aromatic nucleus (compounds **2**, **7**, and **9**) exhibit a 86%, 72% and 81% of inhibitory activity against the growth of the mycelia of *B. cinerea* compared to those acetylated compounds having acetate groups in the same positions which have 76, 64 and 72% mycelial growth inhibition (compounds **6**, **8** and **10**). These results suggest that the activity is mainly determined by the presence of the geranyl chain. However, by comparing the activities of compounds **11** and **10** it can be concluded that the inhibitory effect almost disappears with a double geranyl chain substitution on the triacetylated aromatic nucleus. The activity is partly recovered when two acetyl groups of compound **11** are replaced by two hydroxyl groups in compound **12**. Thus, for double geranyl substituted compounds the activity depends strongly on the presence of hydroxyl groups.

Our results are consistent with previous investigations [23,24,25,26] reporting that molecules with similar structure present high potential antifungal activity (in the range 1.0 to 10.0 µg) against *Cladosporium cladosporioides* and *C. sphaerospermum* [25]. Taken together with our results it could be concluded that addition of a geranyl chain and hydroxyl groups to the aromatic nucleus increases the biological activity of the molecule by at least a factor of two.

## 3. Experimental

### 3.1. General

Unless otherwise stated, all chemical reagents purchased (Merck, Darmstadt, Germany or Aldrich, St. Louis, MO, USA) were of the highest commercially available purity and were used without previous purification. IR spectra were recorded as thin films in a FT-IR Nicolet 6700 spectrometer (Thermo Scientific, San Jose, CA, USA) and frequencies are reported in cm^−1^. Low resolution mass spectra were recorded on an Agilent 5973 spectrometer (Agilent Technologies, Santa Clara, CA, USA) at 70eV ionising voltage in a DB-5 m, 30 m × 0.25 mm × 0.25 µm column, and data are given as *m/z* (% rel. int.). High resolution mass spectra were recorded on an LTQ Orbitrap XL spectrometer (Thermo Scientific, San Jose, CA, USA) by applying a voltage of 1.8 kV in the positive and 1.9 kV in the negative, ionization mode. The spectra were recorded using full scan mode, covering a mass range from *m/z* 100–1,300. The resolution was set to 50,000 and maximum loading time for the ICR cell was set to 250 ms.^1^H, ^13^C, ^13^C DEPT-135, sel. gs1D ^1^H NOESY, gs2D HSQC and gs2D HMBC spectra were recorded in CDCl_3_ solutions and are referenced to the residual peaks of CHCl_3_ at *δ* = 7.26 ppm and *δ* = 77.0 ppm for ^1^H and ^13^C, respectively, on a Bruker Avance 400 Digital NMR spectrometer (Bruker, Rheinstetten, Germany), operating at 400.1 MHz for 1H and 100.6 MHz for ^13^C. Chemical shifts are reported in *δ* ppm and coupling constants (*J*) are given in Hz. Silica gel (Merck 200–300 mesh) was used for C.C. and silica gel plates HF_254_ for TLC. TLC spots were detected by heating after spraying with 25% H_2_SO_4_ in H_2_O.

### 3.2. Synthesis of Geranylphenol Derivatives

*(E)-2-(3,7-Dimethylocta-2,6-dienyl)benzene-1,4-diol (1,4-geranylhydroquinone)* (**2**). To a solution of 1,4-hydroquinone (1.12 g, 10.2 mmol) and geraniol (1.57 g, 10.2 mmol) in acetonitrile (30 mL), saturated with AgNO_3_, was slowly added BF_3_^.^OEt_2_ (0.45 g, 3.2 mmol) dropwise with stirring at room temperature and under a N_2_ atmosphere. After the addition was completed, the stirring was continued for 48 h. The end of the reaction was verified by TLC, and then the mixture was poured onto crushed ice (30 g) and extracted with EtOAc (3 × 20 mL). The organic layer was washed with 5% NaHCO_3_ (30 mL) and water (2 × 15 mL), dried over Na_2_SO_4_, and filtered. The solvent was evaporated under reduced pressure. The crude was re-dissolved in CH_2_Cl_2_ (5 mL) and chromatographed on silica gel with petroleum ether/EtOAc mixtures of increasing polarity (19.8:0.2→14.0:6.0). Compound **2** was obtained as a brownish oil (0.736 g, 32% yield). Spectroscopic data of compound **2** was consistent with those in the literature [12,16,17].

*(E)-2-(3,7-Dimethylocta-2,6-dienyl)-1,4-phenylene diacetate* (**6**). To a solution of compound **2** (0.05 g, 0.45 mmol), DMAP (5.0 mg) in dichloromethane (20 mL) and pyridine (2.0 mL) was added Ac_2_O (0.54 g, 5.3 mmol). The end of the reaction was verified by TLC (0.5 h), and the mixture was extracted with EtOAc (2 × 20 mL). Then the organic layer was washed with 5% KHSO_4_ (2 × 10 mL) and water (2 × 10 mL), dried over Na_2_SO_4_, filtered and the solvent was evaporated under reduced pressure. The crude was re-dissolved in CH_2_Cl_2_ (5 mL) and chromatographed on silica gel with petroleum ether/EtOAc mixtures of increasing polarity (19.8:0.2→16.8:3.2). Compound **6** was obtained as a viscous yellow oil (0.0818 g, 92.8% yield). IR (cm^−1^): 2967; 2917; 1763; 1490; 1368; 1169; 1012; 914. ^1^H-NMR: 7.02 (d, *J* = 9.5 Hz, 1H, H-6); 6.94 (m, 2H, H-3 and H-5); 5.22 (t, *J* = 7.2 Hz, 1H, H-2'); 5.09 (t, *J* = 6.1 Hz, 1H, H-6'); 3.23 (d, *J* = 7.2 Hz, 2H, H-1'); 2.30 (s, 3H, COCH_3_); 2.28 (s, 3H, COCH_3_); 2.09–2.08 (m, 2H, H-5'); 2.06–2.05 (m, 2H, H-4'); 1.68 (s, 3H, H-8'); 1.66 (s, 3H, CH_3_-C3'); 1.60 (s, 3H, CH_3_-C7'). ^13^C-NMR: 169.4 (2 × COCH_3_); 148.3 (C-4); 146.3 (C-1); 137.6 (C-3'); 134.9 (C-2); 131.6 (C-7'); 124.1 (C-6'); 122.9 (C-6); 122.6 (C-3); 120.7 (C-2'); 119.9 (C-5); 39.6 (C-4'); 28.5 (C-1'); 26.5 (C-5'); 25.7 (C-8'); 21.1 (COCH_3_); 20.8 (COCH_3_); 17.7 (CH_3_-C7'); 16.1 (CH_3_-C3').

*(E)-4-(3,7-Dimethylocta-2,6-dienyl)benzene-1,2,3-triol* (**7**). To a solution of pyrogallol (1.25 g, 9.9 mmol) and geraniol (1.8 g, 11.9 mmol) in acetonitrile (30 mL), saturated with AgNO_3_, was slowly added BF_3_^.^OEt_2_ (0.7 g, 4.8 mmol) dropwise with stirring at room temperature and under a N_2_ atmosphere. After the addition was completed, the stirring was continued for 24 h. The end of the reaction was verified by TLC, and then the mixture was poured onto crushed ice (30 g) and it was extracted with EtOAc (3 × 20 mL). The organic layer was washed with 5% NaHCO_3_ (30 mL) and water (2 × 15 mL), dried over Na_2_SO_4_, and filtered. The solvent was evaporated under reduced pressure. The crude product was redissolved in CH_2_Cl_2_ (5 mL) and chromatographed on silica gel with petroleum ether/EtOAc in isocratic mixtures (20:80). Compound **7** was obtained as a reddish oil (0.47 g, 18.1% yield). IR (cm^−1^): 3396; 2966; 2917; 1627; 1472; 1375; 1285; 1027; 794. ^1^H-NMR: 6.53 (d, *J* = 8.3 Hz, 1H, H-5); 6.44 (d, *J* = 8.3 Hz, 1H, H-6); 5.45 (bs, 3H, OH); 5.31 (t, *J* = 7.1 Hz, 1H, H-2'); 5.06 (t, *J* = 5.9 Hz, 1H, H-6'); 3.30 (d, *J* = 7.1 Hz, 2H, H-1'); 2.13–2.11 (m, 2H, H-5'); 2.09–2.06 (m, 2H, H-4'); 1.76 (s, 3H, CH_3_-C3'); 1.68 (s, 3H, H-8'); 1.60 (s, 3H, CH_3_-C7'). ^13^C-NMR: 142.5 (C-3); 142.3 (C-1); 138.4 (C-3'); 132.1 (C-7'); 132.0 (C-2); 123.8 (C-6'); 122.2 (C-2'); 120.2 (C-5); 119.5 (C-4); 107.4 (C-6); 39.6 (C-4'); 29.4 (C-1'); 26.3 (C-5'); 25.7 (C-8'); 17.7 (CH_3_-C7'); 16.1 (CH_3_-C3'). HRMS: (M + 1) calcd. for C_16_H_22_O_3_: 263.1569, found: 263.1547.

*(E)-4-(3,7-Dimethylocta-2,6-dienyl)benzene-1,2,3-triyl triacetate* (**8**). To a solution of compound **7** (0.05 g, 0.129 mmol) and DMAP (3.0 mg) in dichloromethane (20 mL) and pyridine (1.0 mL) was added Ac_2_O (0.54 g, 5.3 mmol). The end of the reaction was verified by TLC (0.5 h), and the mixture was extracted with EtOAc (2 × 20 mL). The organic layer was washed with 5% NaHCO_3_ (30 mL) and water (2 × 15 mL), dried over Na_2_SO_4_, and filtered. The solvent was evaporated under reduced pressure. Then the organic layer was washed with 5% KHSO_4_ (2 × 10 mL) and water (2 × 10 mL), dried over Na_2_SO_4_, filtered and the solvent was evaporated under reduced pressure. The crude was re-dissolved in CH_2_Cl_2_ (5 mL) and chromatographed on silica gel with petroleum ether/EtOAc mixtures of increasing polarity (19.8:0.2→15.4:4.6). Compound **8** was obtained as a viscous yellow oil (0.0686 g, 93% yield). IR (cm^−1^): 2925; 1781; 1491; 1370; 1188; 1045; 871. ^1^H-NMR: 7.12 (d, *J* = 8.6 Hz, 1H, H-5); 7.05 (d, *J* = 8.6 Hz, 1H, H-6); 5.21 (t, *J* = 7.2 Hz, 1H, H-2'); 5.09 (t, *J* = 6.4 Hz, 1H, H-6'); 3.23 (d, *J* = 7.2 Hz, 2H, H-1'); 2.29 (s, 3H, COCH_3_); 2.27 (s, 3H, COCH_3_); 2.26 (s, 3H, COCH_3_); 2.11–2.09 (m, 2H, H-5'); 2.08–2.05 (m, 2H, H-4'); 1.69 (s, 3H, H-8'); 1.66 (s, 3H, CH_3_-C3'); 1.60 (s, 3H, CH_3_-C7'). ^13^C-NMR: 168.0 (COCH_3_); 167.7 (COCH_3_); 167.1 (COCH_3_); 141.7 (C-3); 141.3 (C-2); 137.6 (C-3'); 134.7 (C-1); 132.7 (C-4); 131.6 (C-7'); 126.3 (C-5); 124.1 (C-6'); 120.6 (C-2'); 120.4 (C-6); 39.6 (C-4'); 28.2 (C-1'); 26.4 (C-5'); 25.7 (C-8'); 20.6 (COCH_3_); 20.2 (COCH_3_); 20.1 (COCH_3_); 17.7 (CH_3_-C7'); 16.0 (CH_3_-C3'). HRMS: (M + 1) calcd. for C_22_H_28_O_6_: 389.1886, found: 389.1867.

*(E)-2-(3,7-Dimethylocta-2,6-dienyl)benzene-1,3,5-triol* (**9**). To a solution of phloroglucinol (5.0 g, 39.6 mmol) and geraniol (6.12 g, 39.6 mmol) in acetonitrile (60 mL), saturated with AgNO_3_, was slowly added BF_3_^.^OEt_2_ (1.84 g, 12.8 mmol) dropwise with stirring at room temperature and under a N_2_ atmosphere. After the addition was completed, the stirring was continued for 48 h. The end of the reaction was verified by TLC. The mixture was poured onto crushed ice (30 g) and 20 mL of NaCl (10%) and extracted with EtOAc (3 × 30 mL). Then the organic layer was washed with 5% NaHCO_3_ (30 mL) and water (2 × 20 mL), dried over Na_2_SO_4_, filtered and the solvent was evaporated under reduced pressure. The crude was re-dissolved in CH_2_Cl_2_ (5 mL) and chromatographed on silicagel with petroleum ether/EtOAc mixtures of increasing polarity (19.8:0.2→6.0:14.0). Two fractions were obtained. Fraction I: a complex mixture (630 mg), and Fraction II: Compound **9** (3.30 g, 32% yield) obtained as yellow viscous oil. MS (*m/z*, %): M^+^ 262 (0.2); 191 (100); 175 (52.7); 137 (49.8); 123 (47.8); 69 (22.1). IR (cm^−1^): 3397; 2967; 2925; 1706; 1620; 1515; 1463; 1377. ^1^H-NMR: 5.99 (bs, 2H, OH); 5.93 (s, 2H, H-4 and H-6); 5.22 (t, *J* = 6.8 Hz, 1H, H-2'); 5.03 (t, *J* = 5.9 Hz, 1H, H-6'); 3.30 (d, *J* = 6.8 Hz, 2H, H-1'); 2.08–2.05 (m, 2H, H-5'); 2.03–2.02 (m, 2H, H-4'); 1.76 (s, 3H, CH_3_-C3'); 1.66 (s, 3H, H-8'); 1.57 (s, 3H, CH_3_-C7'). ^13^C-NMR: 155.7 (C-1 and C-3); 154.7 (C-5); 138.7 (C-3'); 131.9 (C-7'); 123.7 (C-6'); 122.0 (C-2'); 106.3 (C-2); 96.07 (C-4 and C-6); 39.6 (C-4'); 26.3 (C-5'); 25.6 (C-8'); 21.9 (C-1'); 17.6 (CH_3_-C3'); 16.0 (CH_3_-C7').

*(E)-2-(3,7-Dimethylocta-2,6-dienyl) benzene-1,3,5-triyl triacetate* (**10**). To a solution of compound **9** (0.109 g, 0.41 mmol) and DMAP (5.0 mg) in dichloromethane (20 mL) and pyridine (2.0 mL) was added Ac_2_O (1.08 g, 10.6 mmol). The end of the reaction was verified by TLC (~1 h), and the mixture was extracted with EtOAc (2 × 25 mL). The organic layer was washed with 5% NaHCO_3_ (30 mL) and water (2 × 15 mL), dried over Na_2_SO_4_, and filtered. The solvent was evaporated under reduced pressure. Then the organic layer was washed with 5% KHSO_4_ (2 × 15 mL) and water (2 × 10 mL), dried over Na_2_SO_4_, filtered and the solvent was evaporated under reduced pressure. The crude was redissolved in CH_2_Cl_2_ (5 mL) and chromatographed on silica gel with petroleum ether/EtOAc mixtures of increasing polarity (19.8:0.2→13.6:6.4). Compound **10** was obtained as a viscous yellow oil (0.138 g, 86.8% yield). MS (*m/z*, %): 240 (27.5); 180 (18.8); 121 (39.8); 120 (64.0); 109 (54.7); 97 (82.3); 82 (49.4); 69 (56.2); 68 (100). IR (cm^−1^): 2969; 2924; 2851; 1775; 1620; 1432. ^1^H-NMR: 6.82 (s, 2H, H-4 and H-6); 5.06 (t, *J* = 6.8 Hz, 1H, H-2'); 5,02 (t, *J* = 5.8 Hz, 1H, H-6'); 3.17 (d, *J* = 6.6 Hz, 2H, H-1'); 2.26 (s, 6H, COCH_3_); 2.24 (s, 3H, COCH_3_); 2.04–2.03 (m, 2H, H-5'); 1.97–1.95 (m, 2H, H-4'); 1.71 (s, 3H, H-8'); 1.66 (s, 3H, CH_3_-C3'); 1.58 (s, 3H, CH_3_-C7'). ^13^C-NMR: 168.6 (COCH_3_); 168.5 (COCH_3_); 149.6 (C-1 and C-3); 148.5 (C-5); 135.9 (C-3'); 131.5 (C-7'); 124.0 (C-2); 124.0 (C-2'); 120.8 (C-6'); 113.8 (C-4 and C-6); 39.5 (C-4'); 26.5 (C-5'); 25.6 (C-8'); 23.6 (C-1'); 21.0 (COCH_3_); 20.8 (COCH_3_); 17.6 (CH_3_-C3'); 16.2 (CH_3_-C7'). HRMS: (M + 1) calcd. for C_22_H_28_O_6_: 389.1886, found: 389.1855.

*2,4-bis((E)-3,7-Dimethylocta-2,6-dienyl)benzene-1,3,5-triyl triacetate* (**11**). To a solution containing Fraction I (630 mg), DMAP (6.0 mg) in dichloromethane (20 mL) and pyridine (2.0 mL) was added Ac_2_O (1.08 g, 10.6 mmol). The end of the reaction was verified by TLC (~1 h), and the mixture was extracted with EtOAc (2 × 25 mL). Then the organic layer was washed with 5% KHSO_4_ (2 × 20 mL) and water (2 × 20 mL), dried over Na_2_SO_4_, filtered and the solvent was evaporated under reduced pressure. The crude was redissolved in CH_2_Cl_2_ (5 mL) and chromatographed on silica gel with petroleum ether/EtOAc in isocratic mixtures (90:10). Compound **11** was obtained as viscous yellow oil (443 mg, 70.3% yield from mixture). IR (cm^−1^): 2968; 2923; 2867; 1772; 1615; 1434. ^1^H-NMR: 6.83 (s, 1H, H-6); 5.06 (t, *J* = 6.8 Hz, 2H, H-6'); 5.02 (t, *J* = 6.5 Hz, 2H, H-2'); 3.12 (bs, 4H, H-1'); 2.26 (s, 3H, COCH_3_); 2.24 (s, 6H, COCH_3_); 2.07–2.02 (m, 4H, H-5'); 1.97–1.94 (m, 4H, H-4'); 1.70 (s, 6H, CH_3_-C3'); 1.66 (s, 6H, H-8'); 1.58 (s, 6H, CH_3_-C7'); ^13^C-NMR: 168.6 (COCH_3_); 168.4 (COCH_3_); 148.6 (C-3); 147.2 (C-1 and C-5); 135.6 (C-3'); 131.3 (C-7'); 124.6 (C-2 and C-4); 124.0 (C-6'); 121.0 (C-2'); 114.9 (C-6); 39.4 (C-4'); 26.4 (C-5'); 25.5 (C-8'); 24.1 (C-1'); 20.7 (COCH_3_); 20.4 (COCH_3_); 17.6 (CH_3_-C7'); 16.1 (CH_3_-C3'). HRMS: (M + 1) calcd. for C_32_H_44_O_6_: 525. 3138, found: 525.3114.

*2,6-bis((E)-3,7-Dimethylocta-2,6-dienyl)-3,5-dihydroxyphenyl acetate* (**12**). To a solution of compound **11** (0.109 g, 0.41 mmol) in methanol (30 mL) was added Et_3_N (0.218 g, 2.15 mmol). Then the reaction mixture was stirred at room temperature for two hours. The end of the reaction was verified by TLC. The solvent was evaporated and the residue resuspended in EtOAc (25 mL). The organic layer was washed with 5% HCl (2 × 10 mL) and water (2 × 20 mL), dried over Na_2_SO_4_, filtered, and the solvent was evaporated under reduced pressure. The crude was redissolved in CH_2_Cl_2_ (5 mL) and chromatographed on silica gel with petroleum ether/EtOAc mixtures of increasing polarity (19.8:0.2→10.6:9.4). Compound **12** was obtained as a viscous yellow oil (0.098 g, 96% yield). IR (cm^−1^): 3446; 2967; 2922; 2856; 1738; 1622; 1446. ^1^H-NMR: 6.16 (s, 1H, H-4); 5.16 (t, *J* = 6.7 Hz, 2H, H2'); 5.05 (t, *J* = 6.5 Hz, 2H, H-6'); 3.14 (bs, 4H, H-1'); 2.30 (s, 3H, COCH_3_); 2.10–2.05(m, 4H, H-5'); 2.02–2.01 (m, 4H, H-4'); 1.75 (s, 6H, CH_3_-C3'); 1.66 (s, 6H, H-8'); 1.58 (s, 6H, CH_3_-C7'); ^13^C-NMR: 169.6 (COCH_3_); 153.8 (C-3 and C-5); 147.8 (C-1); 137.5 (C-3'); 131.8 (C-7'); 123.9 (C-6'); 121.8 (C-2'); 112.2 (C-2 and C-6); 102.4 (C-4); 39.6 (C-4'); 26.4 (C-5'); 25.6 (C-8'); 23.5 (C-1'); 20.6 (COCH_3_); 17.6 (CH_3_-C7'); 16.1 (CH_3_-C3'). HRMS: (M + 1) calcd. for C_28_H_40_O_4_: 441.2927, found: 441.2909.

### 3.3. Fungal Isolate and Culture Condition

In this study, the strain UK of *Botrytis cinerea* was used in all experiments. This strain was isolated from a naturally infected grape (*Vitis vinifera*) and was maintained on potato dextrose agar medium (PDA; Difco, Detroit, MI, USA) at 4 °C. The inoculum of the pathogen was grown on PDA inphotoperiod of 16 h light/8 h dark at 23 °C for 5 days.

### 3.4. Effect of the Compounds on the Mycelial Growth of Botrytis Cinerea *In Vitro*

The anti-phytophatogenic activities of compounds, the negative control (C−) and the positive control (commercial fungicide captan (C+) were assessed using the agar-diffusion assay technique on PDA medium [27]. The compounds were dissolved in ethanol (1%) and water; added at different amounts to obtain a final concentrations of 50, 150 and 250 mg/L in the PDA medium. Negative and positive control experimental conditions for the growth of mycelia of *B. cinerea* were included. Negative control conditions means PDA medium containing 1% ethanol, whereas positive control indicates PDA medium including the commercial fungicide captan at the same concentration specified for the compounds of interest.

A plug (4 mm) of PDA medium with 5-day-old mycelium colonies of the pathogen was placed at the center of a Petri dish with PDA medium with or without compounds of interest. Subsequently, they were incubated under controlled conditions of temperature to 23 °C and photoperiod 16 h light/8 h for 48 h.

The percentage of inhibition was determined for each compounds by expressing the area of *B. cinerea* as a percentage of the negative control. The evaluation was conducted through measuring diameters of mycelial growth after 48 h of incubation. The inhibition percentages of mycelial growth were calculated according to Hou *et al.* [23] for each compound and compared with the negative control. All treatments were performed independently three times in triplicate.

## 4. Conclusions

Linear geranylphenols **2**, **7**, **9** were obtained in yields ranging from 18% to 32% by a previously reported direct Friedel-Craft geranylation reaction. In this work acetonitrile was used as solvent instead of dioxane, and AgNO_3_ was used as secondary catalyst. Under these conditions the yield of **9** was increased from 4% to 32% and a disubstituted coupling product is formed. It seems that AgNO_3_ is able to stabilize the geraniol in CH_3_CN solution. The acetylated derivatives **6**, **8**, **10**, **11** were obtained with yields over 70% by standard acetylation reactions with Ac_2_O and DMAP in CH_2_Cl_2_.

The evaluation of biological activity of geranylphenol derivatives on the growth of mycelia of *B. cinerea* was determined *in vitro* after 48 h of incubation by using an agar-diffusion assay technique with PDA medium. The results show that all compounds, excepting **11**, inhibit its growth between 56%–86% compared to the negative control, at concentrations between 150 to 250 mg/L. The antifungal activity depends on the chemical structure of the geranylphenol derivatives and is mainly determined by the presence of the geranyl chain. Interestingly, the activity is strongly reduced when the aromatic nucleus is substituted with two geranyl chains. Finally, the activity of these compounds is also enhanced by the presence of hydroxyl groups, which is in line with results reported by other authors for similar systems.

## References

[1] Latorre B.A., Flores V., Sara A.M., Roco A. (1994). Dicarboximide-resistant isolates of *Botrytis cinerea* from table grape in Chile-survey and characterization. Plant Dis..

[2] Wedge D.E., Camper N.D., Cutler H.G., Cutler S.J. (2000). Agrochemicals and Pharmaceuticals. Biologically Active Natural Products.

[3] Fenical W. (1974). 4th Proceeding of Food Drugs from the Sea.

[4] Lin H.C., Chang W.L. (1991). Phytochemical and pharmacological study on *Salvia miltiorihiza* (I) isolation of new tanshinones. Chin. Pharm..

[5] Manners G.D. (1977). The hydroquinone terpenoids of *Cordia alliodora*. J. Chem. Soc. Perkin Trans. 1.

[6] Reynolds G., Rodriguez E. (1979). Geranylhydroquinone: A contact allergen from trichomes of Phaceliacrenulata. Phytochemistry.

[7] Reynolds G., Epstein W.L., Terry D., Rodriguez E. (1980). A potent contact allergen of *Phacelia* (Hydrophyllaceae). Contact Derm..

[8] Reynolds G., Rodriguez E. (1981). Prenylated phenols that cause contact dermatitis from trichomes of Phaceliaixodes. Planta Med..

[9] Sato A., Shindo T., Kasanuki N., Hasegawa K. (1989). Antioxidant metabolites from the tunicate *Amaroucium multiplicatum*. J. Nat. Prod..

[10] Benslimane A.F., Pouchus Y.F., Leboterff J., Verbist J.F., Roussakis C., Monniot F. (1988). Cytotoxic and antibacterial substances from the ascidian *Aplidium antillense*. J. Nat. Prod..

[11] De Rosa S., de Giulio A., Iodice C. (1994). Biological effects of prenylated hydroquinones: Structure-activity relationship studies in antimicrobial, brine shrimp, and fish lethality assays. J. Nat. Prod..

[12] Rudali G., Menetrier L. (1967). Action de la géranyl-hydroquinone sur différents cancers spontanés et provoqués chez celles souris. Therapie.

[13] Rudali G. (1966). Research on the radioprotective action of geranyl-hydroquinone. C. R. Seances Soc. Biol. Ses. Fil..

[14] Rodriguez E., Hedin P. (1983). Cytotoxic and Insecticidal Chemicals of Desert Plants. Plant Resistance to Insects.

[15] Sladic D., Gasic M.J. (2006). Reactivity and biological activity of the marine sesquiterpene hydroquinone avarol and related compounds from sponges of the order *Dictyoceratida*. Molecules.

[16] Baeza E., Catalan K., Pena-Cortes H., Espinoza L., Villena J., Carrasco H. (2012). Synthesis of geranylhydroquinone derivatives with potential cytotoxic activity. Quim. Nova.

[17] Baeza E., Catalan K., Villena J., Carrasco H., Cuellar M., Espinoza L. (2012). Synthesis and cytotoxic activity of geranylmethoxyhydroquinone derivatives. J. Chil. Chem. Soc..

[18] Taborga L., Vergara A., Osorio M., Carvajal M., Madrid A., Marilaf F., Carrasco H., Espinoza L. (2013). Synthesis and NMR structure determination of new linear geranylphenols by direct geranylation of activated phenols. J. Chil. Chem. Soc..

[19] Takenaka K., Tanigaki Y., Patil M.L., Rao C.V.L., Takizawa S., Suzuki T., Sasai H. (2010). Enantioselective 6-endo-trig Wacker-type cyclization of 2-geranylphenols: Application to a facile synthesis of (−)-cordiachromene. Tetrahedron Asymmetry.

[20] Inouye H., Tokura K., Tohita S. (1968). Uber die Inhaltsstoffe von Pirolaceen. XV Zur Struktur des Pirolatins. Chem. Ber..

[21] Raikar S.B., Nuhant P., Delpech B., Marazano C. (2008). Synthesis of polyprenylated benzoylphloroglucinols by regioselective prenylation of phloroglucinol in an aqueous medium. Europ. J. Org. Chem..

[22] Fedorov S.N., Radchenko O.S., Shubina L.K., Balaneva N.N., Bode A.M., Stonik V.A., Dong Z.G. (2006). Evaluation of cancer-preventive activity and structure-activity relationships of 3-demethylubiquinone Q2, isolated from the ascidian *Aplidium glabrum*, and its synthetic analogs. Pharm. Res..

[23] Hou Z., Yang R., Zhang C., Zhu L., Miao F., Yang X., Zhou L. (2013). 2-(Substituted phenyl)-3,4-dihydroisoquinolin-2-iums as novel antifungal lead compounds: Biological evaluation and structure-activity relationships. Molecules.

[24] Danelutte A.P., Lago J.H.G., Young M.C.M., Kato M.J. (2003). Antifungal flavanones and prenylated hydroquinones from Piper crassinervium Kunth. Phytochemistry.

[25] Lago J.H.G., Ramos C.S., Casanova D.C.C., Morandim A.D., Bergamo D.C.B., Cavalheiro A.J., Bolzani V.D., Furlan M., Guimaraes E.F., Young M.C.M. (2004). Benzoic acid derivatives from piper species and their fungitoxic activity against *Cladosporium cladosporioides* and *C. sphaerospermum*. J. Nat. Prod..

[26] Malami I. (2012). Prenylated benzoica acid derivates from piper species as source of anti-infective agents. Int. J. Pharm. Sci. Res..

[27] Mendoza L., Espinoza P., Urzua A., Vivanco M., Cotoras M. (2009). *In vitro* antifungal activity of the diterpenoid 7á-hydroxy-8(17)-labden-15-oic acid and its derivatives against *Botrytis cinerea*. Molecules.

